# Practical Points

**Published:** 1897-06-26

**Authors:** 


### PRACTICAL POINTS.

*The Expenditure of Hospitals having a Convalescent Home Attached.*—"*Hospital Secretary*" *writes*: "*The cost of our convalescent home is £1,369. This does not include any charge for administration. Therefore the percentage of administration for the hospital alone is unduly swollen because we supply our convalescent home with income and administration. The percentage of administration on the expenditure of the hospital, including the expenditure on the convalescent home, is 11 per cent., but if the expenditure on the hospital alone be taken it amounts to 12 per cent. I should be glad to learn how to deal with this point.*"

It seems to us the point raised is easy of solution. Before the days of convalescent institutions attached to individual hospitals, it was customary for the hospitals to subscribe or pay so much for patients sent from the hospital to convalescent institutions, and that sum was entered in the ordinary income and expenditure account as an expenditure upon the patients necessary to complete their recovery. We think, therefore, that the expenditure on the convalescent institution should be embodied in the income and expenditure account of the hospital, the items being distributed in the second column under the analysed heads, and carried out totals. Such a course, whilst keeping the expenditure of the convalescent institution distinct, would show in one account the whole expenditure of the hospital upon patients, and so secure an accurate calculation as to the percentage of the cost of administration on the one hand, and on the other prevent any chance of excess of expenditure in the convalescent institution, because that expenditure will be clearly shown as a separate item, though embodied in the totals of the income and expenditure account.

